# Influence of prenatal hypoxia and postnatal hyperoxia on morphologic lung maturation in mice

**DOI:** 10.1371/journal.pone.0175804

**Published:** 2017-04-20

**Authors:** Andreas Schmiedl, Torge Roolfs, Erol Tutdibi, Ludwig Gortner, Dominik Monz

**Affiliations:** 1 Institute of Functional and Applied Anatomy, Hannover Medical School, Hannover, Germany; 2 Biomedical Research in Endstage und Obstructive Lung Disease Hannover (BREATH), Member of the German Center for Lung Research (DZL), Hannover Medical School, Hannover, Germany; 3 REBIRTH Cluster of Excellence, Hannover Medical School, Hannover, Germany; 4 Department of Pediatrics and Neonatology, Saarland University, Homburg/Saar, Germany; Emory University School of Medicine, UNITED STATES

## Abstract

**Background:**

Oxygen supply as a lifesaving intervention is frequently used to treat preterm infants suffering additionally from possible prenatal or perinatal pathogen features. The impact of oxygen and/or physical lung injury may influence the morphological lung development, leading to a chronic postnatal lung disease called bronchopulmonary dysplasia (BPD). At present different experimental BPD models are used. However, there are no systematic comparative studies regarding different influences of oxygen on morphological lung maturation.

**Objective:**

We investigated the influence of prenatal hypoxia and/or postnatal hyperoxia on morphological lung maturation based on stereological parameters, to find out which model best reflects morphological changes in lung development comparable with alterations found in BPD.

**Methods:**

Pregnant mice were exposed to normoxia, the offspring to normoxia (No/No) or to hyperoxia (No/Hyper). Furthermore, pregnant mice were exposed to hypoxia and the offspring to normoxia (Hypo/No) or to hyperoxia (Hypo/Hyper). Stereological investigations were performed on all pups at 14 days after birth.

**Results:**

Compared to controls (No/No) 1) the lung volume was significantly reduced in the No/Hyper and Hypo/Hyper groups, 2) the volume weighted mean volume of the parenchymal airspaces was significantly higher in the Hypo/Hyper group, 3) the total air space volume was significantly lower in the No/Hyper and Hypo/Hyper groups, 4) the total septal surface showed significantly lower values in the No/Hyper and Hypo/Hyper groups, 5) the wall thickness of septa showed the highest values in the Hypo/Hyper group without reaching significance, 6) the volume density and the volume weighted mean volume of lamellar bodies in alveolar epithelial cells type II (AEII) were significantly lower in the Hypo/Hyper group.

**Conclusion:**

Prenatal hypoxia and postnatal hyperoxia differentially influence the maturation of lung parenchyma. In 14 day old mice a significant retardation of morphological lung development leading to BPD-like alterations indicated by different parameters was only seen after hypoxia and hyperoxia.

## Introduction

During prenatal lung development an embryonic stage and a fetal stage can be distinguished in all vertebrates. The fetal stage is subdivided into three morphologically differentiated stages [1–4]. The pseudoglandular stage (human lung 7^th^-15^th^ week of gestation (gw); mouse lung: 9^th^-16^th^ gestational day (gd)) is characterized by growing and subdividing of the future conductive bronchial tree up to the terminal bronchioles. The canalicular stage (human lung: 16^th^-26^th^ gw; mouse lung: 16^th^-18^th^ gd) represents the formation of the lung acinus, the channeling of the mesenchyme by capillaries, the differentiation of the alveolar epithelial cells and the first respiratory sections. In the saccular stage (human lung: 24^th^ gw until birth; mouse lung: 18^th^ gd until 3^rd^ postnatal day) there is formation of sacculi separated by more or less thick septa containing much connective tissue and a double layered capillary bed. The alveolar stage already starts in humans in the 36^th^ gw followed by the stage of vascular maturation leading to a monolayered capillary bed. In humans 80–85% of alveoli are formed after birth [5]. The postnatal alveolarization phase lasts at least until young adulthood [6–8]. During alveolarization the alveoli are formed by developing secundary septa. In mouse lungs, the alveolar stage begins postnatally on day 4 after birth and lasts nearly 10 days [3,9].

Already in the canalicular phase alveolar epithelial cells type II (AEII) start the synthesis of surface active agent (surfactant) and form inclusions, the later lamellar bodies (Lb) as surfactant storage organelles. Even small quantities of secreted surfactant are detectable [1,10]. Pulmonary surfactant is a complex of phospholipids and surfactant proteins (SP) spreading at the air liquid interface of alveoli and small bronchi to prevent collapsing [11]. The intracellular pool is localized in the AEII, which synthetize, store, secret and reuptake surfactant [12]. The intra-alveolar surfactant consists of the surface bilayer as well as of surface active and inactive subtypes located in the hypophase [13]. The mature hydrophobic SP-B and SP-C are mainly found in Lb and are integrated in the phospholipid bilayer after secretion [14]. The immunomodulating hydrophilic SP-A and SP-D are predominantly stored in the multivesicular bodies and are secreted independent of the Lb [15].

In contrast to the human lung, the rodent lung is morphologically immature at birth comparable with the saccular stage of preterm infants. Therefore rodents are suitable for studying perinatal lung development. In perinatal care, birth of premature infants with a gestational age between 23 and 27 weeks leads to considerable complications influencing neonatal mortality [16,17]. Such preterm, very low birth weight infants are at risk to develop a chronic respiratory disease called bronchopulmonary dysplasia (BPD) [17–19]. Besides immaturity, many other factors such as surfactant deficiency/dysfunction, inflammation, infection, nutrition, mechanical ventilation, genetics, oxygen and / or intrauterine environment can contribute to the development of BPD [18,20]. The occurrence of parenchymal fibrosis, edema, vascular changes and persistent inflammation in lungs originally characterized BPD [17,19,21,22]. Progress in neonatal care ameliorates the survival of infants with very low birth weight with the risk of developing a “new” BPD. Morphological characteristics of the ‘new BPD’ are fewer and larger alveoli as a result of interrupted septation and abnormal vascular organization [19,21,23].

Various animal models have been used for studying BPD. Predominantly prenatal hypoxia or LPS application and/or postnatal hyperoxia were induced and the BPD-like alterations were determined by different stereological parameters [23–28]. Alterations in pre- and / or postnatal oxygen concentrations may additionally influence the surfactant system [29]. Depending on the timing of the insult, acceleration or deceleration of surfactant development may occur [30]. Also postnatal hyperoxia influences the biosynthesis and secretion of surfactant in differential ways [31–33].

This study was carried out to compare the influence of prenatal hypoxia and / or postnatal hyperoxia on morphological lung maturation. Using established animal models and standardized stereological parameters, the aim of this study was to find out which model best reflects morphological changes in lung development comparable with alterations found in BPD.

## Materials and methods

### Animal model

Animal maintenance and the experiments were carried out in accordance with the European Communities Council Directive of 24 November 1986 (86/609/EEC) and were approved by the local board for animal welfare (Landesamt für Gesundheit und Verbraucherschutz Abteilung Lebensmittel und Veterinärwesen, Saarbrücken, Germany, AZ: H-1 2.4.2.2). Conduct of the animal experiments followed the ARRIVE guidelines whenever possible [34].

**Fig 1:**
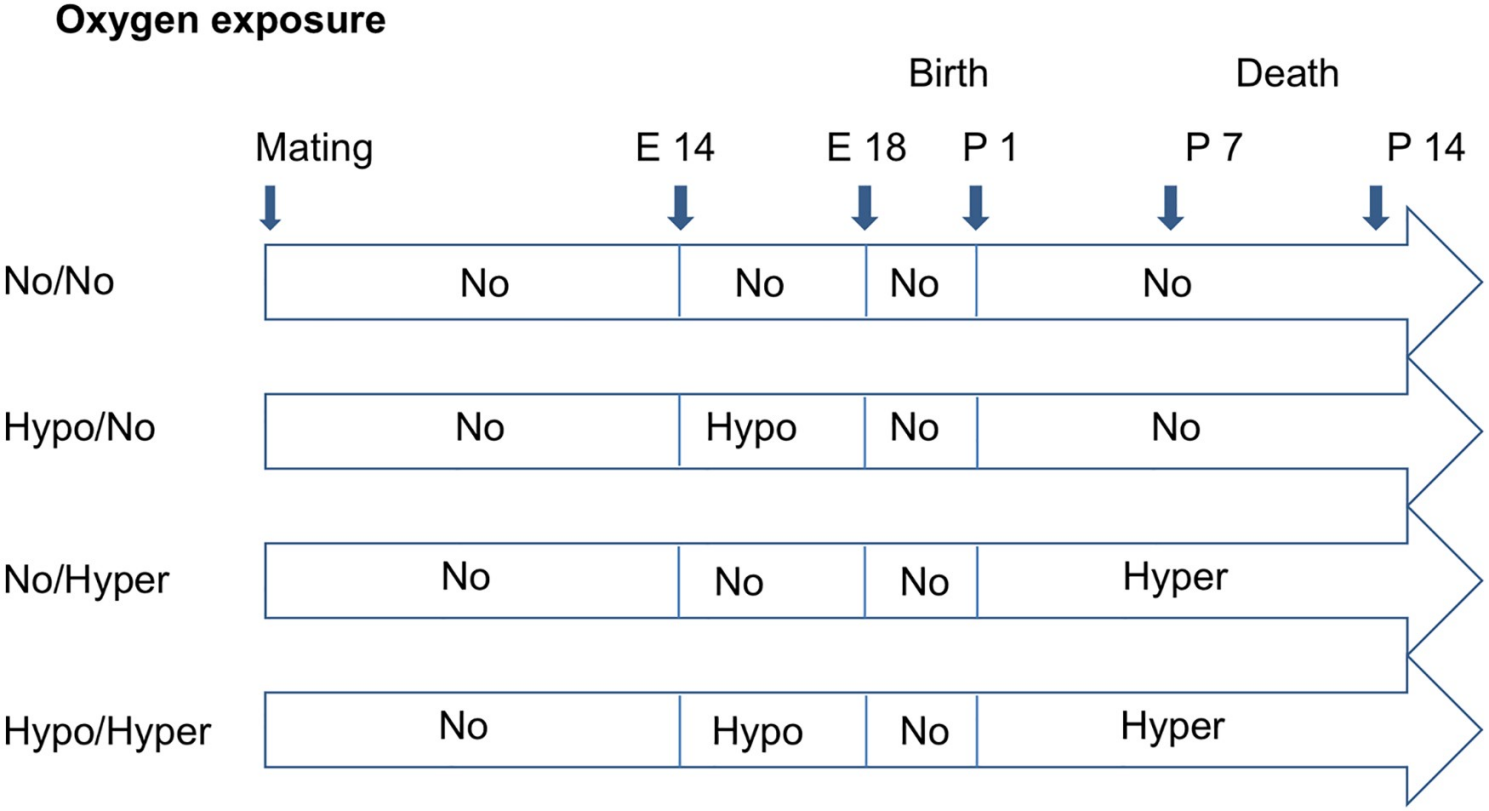
Design of experimental procedure. No/No = normoxic control group (FiO_2_ = 0.21); Hypo/No = prenatal hypoxia (FiO_2_ = 0.10) and postnatal normoxia; No/Hyper = prenatal normoxia and postnatal hyperoxia (FiO_2_ = 0.75); Hypo/Hyper = prenatal hypoxia and postnatal hyperoxia.

Pregnant mice (C57BL/6, Charles River, Sulzfeld, Germany) were kept with ad libitum access to food and water. At gestational day 14, the dams were randomly divided into groups (Fig 1).

In the control group the dams and their pups remained under normoxic conditions (normoxia, fraction of inspired oxygen, FiO_2_ = 0.21; No/No group).

In the Hypo/No group, the pregnant dams were put into ventilated chambers, in which hypoxia (FiO_2_ = 0.10) was induced by introducing nitrogen into the chamber to induce fetal growth restriction from gestational day 14 until gestational day 18, as described before [35]. After birth the pups were kept under normoxic conditions.

In the No/Hyper group, the dams were housed under normoxia. After birth the dams and their pubs were exposed to hyperoxia (FiO_2_ = 0.75) in the ventilated chamber. Oxygen was introduced into the chamber starting from postnatal day 1 to postnatal day 14 to induce lung injury.

The Hypo/Hyper group included dams exposed to hypoxia from gestational day 14 to 18 (FiO_2_ 0.10) and after birth with their pups to hyperoxia (FiO_2_ 0.75) from postnatal day 1 to 14 [36].

### Lung fixation

14 day old pups of the different groups were sacrificed by an intraperitoneal injection of 400 mg pentobarbital per kg body weight. The heart lung block was immediately removed and the lungs were fixed with 1% paraformaldehyde + 1% glutaraldehyde in 0.1 M cacodylate buffer by tracheal instillation with a pressure of 20 cm liquid column using an instillation device as described before [25,36]. After fixation connective tissue and the heart were removed and the volume of the lungs was determined according to the fluid displacement method [37,38].

### Sampling and tissue processing

Sampling and processing of the lungs were carried out as described elsewhere [25,38,39]. Briefly, each lung was embedded in 2% aqueous agar—agar (Merck, Darmstadt, Germany) and cut from apex to base into parallel slices with a thickness of 2 mm using a tissue slicer. Slices from both lungs were alternatively taken for light and electron microscopy. Lung slices collected for electron microscopy were cut into tissue blocks with a size of 1–2 mm^3^. After additional immersion fixation, specimens for light and electron microscopy were rinsed repeatedly in cacodylate buffer. Postfixation followed in 1% OSO_4_ in 0.1 M cacodylate buffer for 2 h. Again specimens were rinsed in cacodylate buffer then in distilled water and stained en bloc overnight at 4–8°C in half-saturated aqueous uranyl acetate solution. Specimens were dehydrated in ascending series of acetone. For ultrastructural analyses tissue blocks were embedded in epoxy resin (Serva Electrophoresis GmbH, Heidelberg, Germany). Prepared 70 nm thin ultra-thin sections were stained with lead citrate and uranyl acetate. For light microscopy whole tissue slices were embedded in the methacrylate resin Technovit 7100 (Heraeus Kulzer GmbH, Hanau, Germany). 1.5 μm sections were cut and stained with toluidine blue.

### Stereological parameters

Lungs were analyzed using an Axioscope light microscope (Zeiss, Oberkochen, Germany) equipped with a computer-assisted stereology tool box (Cast 2.0; Olympus, Ballerup, Denmark).

Using a multipurpose test system stereological methods were performed on the light and electron microscopic level according to the guidelines for quantitative assessment of lung structure [40]. Alveolar epithelial cells type II (AEII) were collected according to the systematic random sampling [41] using a transmission electron microscope (TEM) (Morgagni II 268, Fa. FFEI, Oregon USA) provided with a digital camera (Veleta CCD, Olympus SIS, Münster, Germany). Stereological evaluation was performed using the software Stepanizer^®^stereology tool Version 1 [42].

The following parameters were determined using the point (P) and intersection (IS) counting method [43,44].

### Light microscopy

The volume densities of septa and terminal parenchymal air spaces (V_V_(septa, par), V_V_(airspace, par)) were evaluated to get information about the degree of transition from the canalicular into the saccular phase combined with reduced connective tissue and increased terminal air spaces.

The septal surface densities (S_V_(septa, par)) serve as a parameter for septation and therefore indirectly for alveolarization. The lower the S_V_(septa, par) the lesser is the formation of secondary septa and the lesser is the number of alveoli and the higher is the size of airspaces. The volume to surface ratio of septa (V_S_-ratio_septa_) is a parameter for the septal wall thickness. The thicker the septal wall the more connective tissue is within the septa and the more immature are the septa.

$$V_{V}\left(septa,par\right)\left(\%\right)=\frac{P_{septa}}{P_{par}}\times 100$$

$$V_{V}\left(airspace,par\right)\left(\%\right)=\frac{P_{terminal\;airspaces}}{P_{par}}\times 100$$

$$S_{V}\left(septa,par\right)\left(\frac{1}{\mu m}\right)=4\;\times \;\frac{IS_{septa}}{P_{par}}\times \;L\;\left(length\;of\;test\;lines\right)$$

$$V_{S}-ratio_{septa}\left(mean\;wall\;thickness;\mu m\right)=P_{septa}\;\times \;\frac{L}{4}\times \;IS_{septa}$$

The volume weighted mean volume of parenchymal air space was used for studying changes in the alveolar, saccular and ductal volume as well as their volume distribution and determined using the point sample intercept method [39,40,45,46] using the following formula: $\nabla_{V}\left(\mu m^{3}\right)=\frac{\pi }{3}\times \;I^{3}$ *(I*^*3*^ = *mean of the cubed point sampled intercepts with alveoli*, *sacculi*, *ductus)*.

### Electron microscopy

The V_V_ of AEII and Lb promote some information on the portion of AEII within the septa and the portion of Lb within the AEII and is therefore a morphological correlate of surfactant synthesis.

$$V_{V}\left(AEII,\;septa\right)\left(\%\right)=\frac{P_{AEII}}{P_{septa}}\times \;100$$

$$V_{V}\left(Lb,\;AEII\right)\left(\%\right)=\frac{P_{Lb}}{P_{AEII}}\times \;100$$

Using additionally the point sample intercept method [39,40] the volume weighted mean volume of AEII and Lb was determined by the following formula: $\nabla_{V}\left(\mu m^{3}\right)=\frac{\pi }{3}\times \;I^{3}$ *(I*^*3*^ = *mean of the cubed point sampled intercepts of or AEII or Lb)*.

V_V_ and S_V_ are parameters which are related to the reference space (par) [40]. The reference space could be influenced by alterations such as fluid shifts or shrinkage leading to a reference trap [47,48]. Therefore, we applied both parameters to the total lung volume using the following formula:

$$V\left(septa,lung\right)\;\left(cm^{3}\right)=V_{V}\left(septa,\;par\right)\;\times \;V_{V}\left(par,\;par+nonpar\right)\times \;V_{lung}$$

$$V\left(airspace,lung\right)\;\left(cm^{3}\right)=V_{V}\left(airspace,\;par\right)\;\times \;V_{V}\left(par,\;par+nonpar\right)\times \;V_{lung}$$

$$S\left(septa,lung\right)\;\left(cm^{2}\right)=S_{V}\left(septa,\;par\right)\;\times \;V_{V}\left(par,\;par+nonpar\right)\times \;V_{lung}$$

$$V\left(AEII,lung\right)\;\left(cm^{3}\right)=V_{V}\left(AEII,\;septa\right)\;\times \;V_{V}\left(septa,\;par\right)\;\times \;V_{V}\left(par,\;par+nonpar\right)\times \;V_{lung}$$

$$V\left(Lb,lung\right)\left(cm^{3}\right)=V_{V}\left(Lb,\;AEII\right)\;\times \;V_{V}\left(AEII,\;septa\right)\;\times \;V_{V}\left(septa,\;par\right)\;\times \;V_{V}\left(par,\;par+nonpar\right)\times \;V_{lung}$$

Because complete lungs were not available from all experimental groups, but often only the right or the left lung, the total volumes of lung, airspace, septa and Lb related to left, right or both lungs were presented as relative percentage of the mean control values to ensure comparability.

### Statistical analysis

All treatment values were presented as means ± SD of experiment-specific controls unless otherwise stated. To evaluate the results for their statistical significance the One-Way ANOVA test was used for normally distributed values. For not normally distributed values the nonparametric Kruskal Wallis test was used. Multiple comparisons were corrected with the Dunnett´s multiple comparison test. A level of p<0.05 was considered significant. The GraphPad Prism 6.0 (Statcon, Witzenhausen, Germany) was used.

## Results

### Lung volume

The lung volumes of the 14 day old mice were determined using the fluid displacement method. The mean total volume of both lungs amounted to 253±17μl in the control lungs (n = 8). The lung volumes differed significantly in the investigated groups (p<0.002). Compared to controls (lung volume = 100%) in the Hypo/No (n = 3) group lung volume amounted to 78±2%, in the No/Hyper group (n = 5) to 56±4% (p<0.05) and in the Hypo/Hyper group (n = 7) to 66±18% (p<0.05) (Table 1).

**Table 1:** Total volumes given in percent of controls.

Group	No/No (n = 10)	Hypo/No (n = 3)	No/Hyper (n = 5)	Hypo/Hyper (N = 7)
V(lung) (%)	100±6	78±2	56±4*	66±18*
V(airspace, lung) (%)	100±13	76±13	55±9*	61±27*
V(septa, lung) (%)	98±32	95±42	48±26	83±44
S(septa, lung) (%)	102±14	84±2	39±7*	48±16*
V(AEII, lung) (%)	98±32	96±33	48±20	82±39
V(Lb, lung) (%)	95±40	80±25	44±28	50±26

The total volumes (means ± SD) of lung, airspace, septa, alveolar epithelial cells type II (AEII) and lamellar bodies (Lb) related to left, right or both lungs were presented as relative percentage of the mean control values to ensure comparability. No/No = normoxic control group (FiO_2_ = 0.21); Hypo/No = prenatal hypoxia (FiO_2_ = 0.10), postnatal normoxia; No/Hyper = prenatal normoxia and postnatal hyperoxia (FiO_2_ = 0.75); Hypo/Hyper = prenatal hypoxia and postnatal hyperoxia;

*p<0.05 compared to controls

### Lung morphology

**Fig 2:**
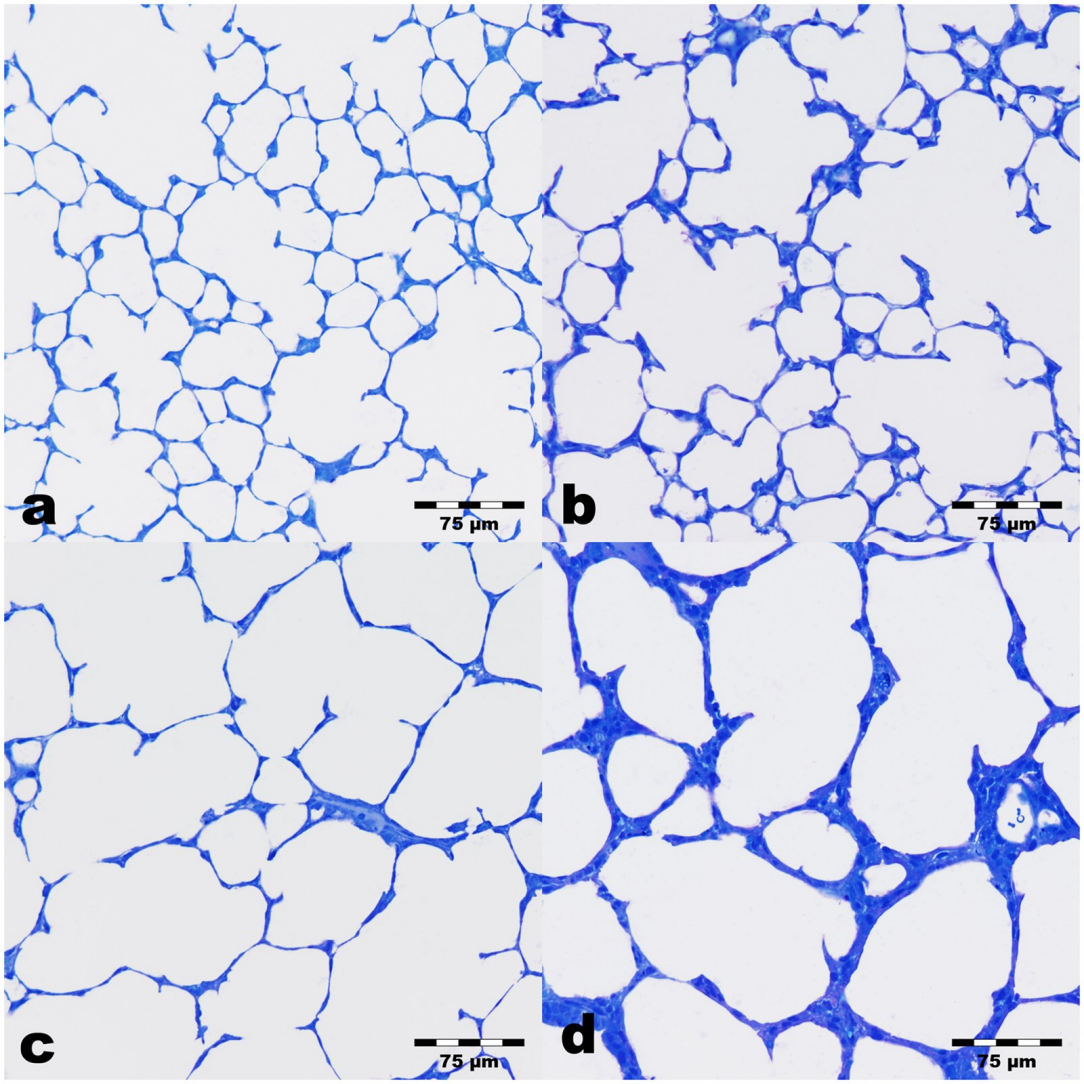
Lung parenchyma of 14 day old mice. Sections were stained with toluidine blue. a) Control lungs (No/No) exhibit well developed formed septa, alveoli and ductus alveolares. b) Prenatal hypoxia induced lungs (Hypo/No) show lung parenchyma without clearly visible alterations compared to controls. c) Postnatal hyperoxia induced lungs (No/Hyper) indicate more expanded airspaces with fewer septa than controls. d) Lungs exposed to prenatal hypoxia and postnatal hyperoxia (Hypo/Hyper) display enlarged parenchymal airspaces surrounded by more or less thick septa.

Looking at the light microscopic level, the control lungs exhibited a well developed lung parenchyma corresponding to the postnatal developmental stage of two weeks (Fig 2a). Well pronounced alveoli, ductus alveolares and septa with the still existing two layered capillary bed were seen. In the experimental groups (Fig 2b–2c) lung parenchyma looked more inhomogeneous. The size of partly immature terminal airspaces (as it is not possible to distinguish sacculi and alveoli in two-dimensional sections), the thickness of septa, and the degree of collapsed terminal airspaces differed more or less depending on the oxygen supply. Furthermore, several terminal airspaces showed a large lumen surrounded by thick septa. The ductus are partly expanded in the experimental groups. The differentiation between ductus and terminal airspaces was partly difficult. In the Hypo/No group airspaces with sizes comparable to those of controls (Fig 2a) were predominantly seen. Enlarged sacculi/alveoli were found rarely. The ductus alveolares seemed partly expanded (Fig 2b). In the No/Hyper group enlarged parenchymal airspaces were found more frequently than in the Hypo/No group. However, between more or less enlarged terminal airspaces those with age-related size were visible (Fig 2c). The inhomogeneous degree of maturation in the Hypo/Hyper group was very obvious, too. Predominantly parenchymal areas with large air spaces and more rarely normal septa were found and changed with areas containing very thick septa and small or large airspaces. A representative image is shown in Fig 2d.

### Stereology

Using the point and intersection counting, different volume densities and surface densities were determined to elucidate the degree of lung maturation in 14 day old mice in the different experimental groups. Both volume densities and surface densities are relative parameters. The values could also be influenced by possible alterations within the reference space, the lung parenchyma. Therefore, the densities were additionally related to the lung volume and presented as percentage of controls.

#### Volume densities of parenchymal airspaces and septa were comparable, but total volume of airspace differed partially compared to controls

**Fig 3:**
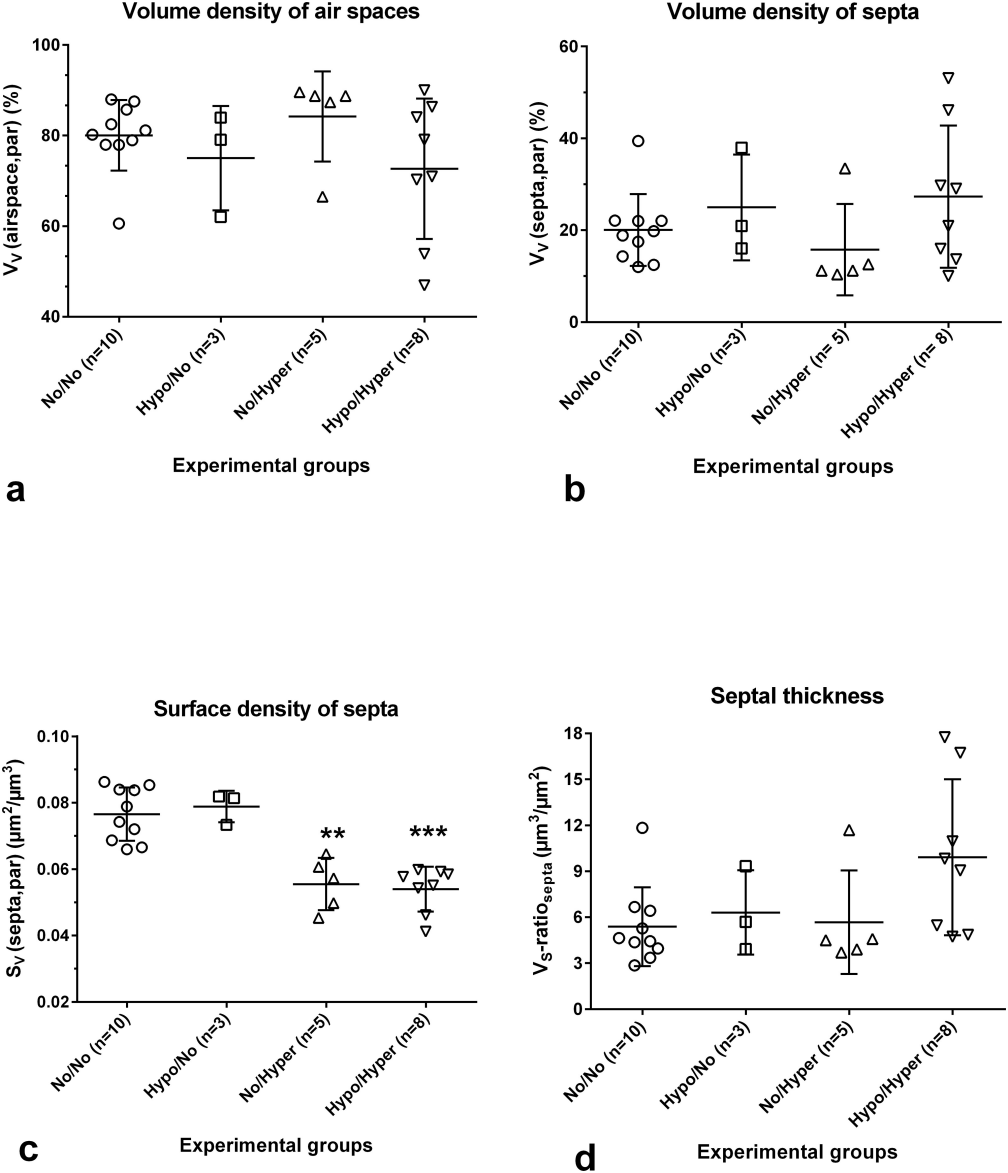
Stereological parameters, that characterize lung parenchyma. Controls (normoxia group, No/No), prenatal hypoxia and postnatal normoxia (Hypo/No group), prenatal normoxia and postnatal hyperoxia ((No/Hyper group) and prenatal hypoxia and postnatal hyperoxia (Hypo/Hyper group). Mean ± SD, *p<0.05, **p<0.01, ***p<0.001 compared to controls. a) The volume densities of airspaces are comparable in all groups. The greatest variation was found in the Hypo/Hyper group. b) The volume densities of alveolar septa are comparable in all groups. The greatest variation was found in the Hypo/Hyper group. c) The septal surface density was significantly reduced in both Hyperoxia groups. d) The mean wall thickness of septa shows a tendency to significance after prenatal hypoxia and postnatal hyperoxia. The values of the other groups are comparable.

The volume density of the parenchymal airspace (alveoli, saccules, ductus) (V_V_(airspace, par)) amounted to 80.06±7.80% (Fig 3a) and the volume density of septa (V_V_(septa, par)) to 20.05±7.83% in the control group (Fig 3b). In the experimental groups values of both parameters showed no differences compared to controls, because these are relative parameters, which influence each other and are further influenced by the reference space (Fig 3a and 3b). The total volume of parenchymal airspace (V(airspace, lung)) differed significantly in the groups investigated (Table 1). Compared to the controls (both lungs, 178±24mm^3^), the V(airspace, lung) was reduced by 45% in the No/Hyper group (n = 3) (p<0.002) and by 39% in the Hypo/Hyper group (n = 7) (p<0.05). The septal volume per lung (V(septa, lung)) showed no group-specific differences (Table 1).

**Fig 4:**
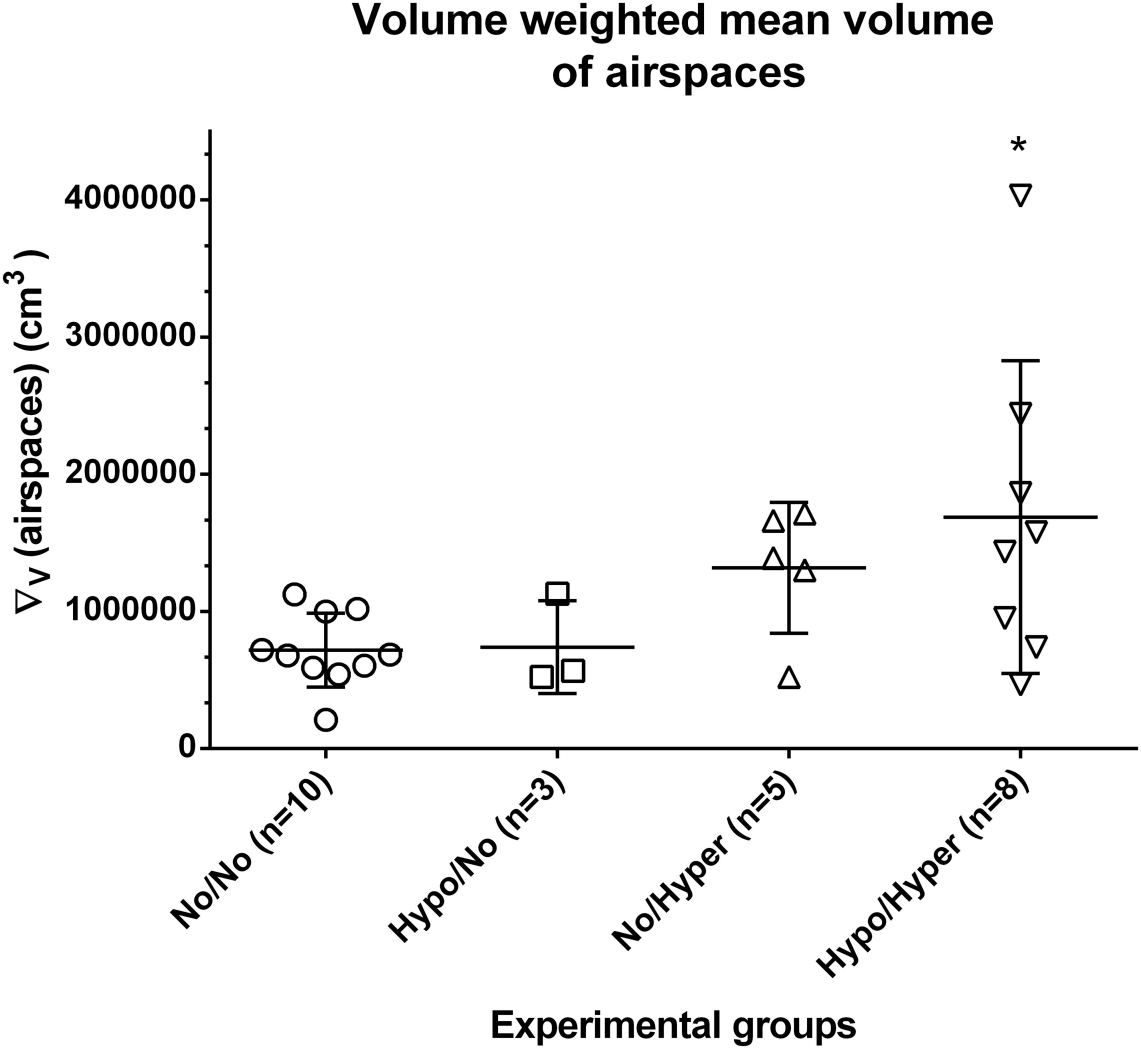
The volume weighted mean volume of airspaces. In controls (normoxia group, No/No), after prenatal hypoxia and postnatal normoxia (Hypo/No group), after prenatal normoxia and postnatal hyperoxia (No/Hyper group) and after prenatal hypoxia and postnatal hyperoxia (Hypo/Hyper group). *p<0.05 compared to controls.

The volume weighted mean volume of parenchymal air spaces (∇_V_(airspace)) was 0.718x10^6^ μm^3^ in controls (Fig 4). While values in the Hypo/No group were comparable with those of controls, values determined in the No/Hyper group showed higher, but not significant values (p<0.13). Only in the Hypo/Hyper group significantly increased values were found (p<0.05). The partly high variances reflect the inhomogeneities of this parameter in the experimental groups (Fig 4).

#### Surface densities as well as total surface area of septa were significantly decreased after postnatal hyperoxia

In controls, the S_V_(septa, par) was 0.077±0.008μm^2^/μm^3^ (Fig 3c). There were significant differences between the different groups (p<0.0003). Multiple comparisons revealed significantly decreased values of S_V_(septa, par) in lungs of the No/Hyper group as well as of the Hypo/Hyper group compared to controls (Fig 3c). However, the S_V_(septa, par) in lungs of the Hypo/No group was comparable with control values (Fig 3c).

The total septal surface area (S(septa, lung)) also differed between the groups (p<0006). Compared to controls (both lungs, 167±24cm^2^), lungs of the Hypo/No group showed comparable values (Table 1). However, the total septal surface in lungs of the No/Hyper group was significantly reduced by 61%. Also values obtained from lungs of the Hypo/Hyper group were significantly reduced by 52% (Table 1).

The V_S_-ratio_septa_, a parameter for the septal thickness, amounted to 5.38±2.57μm. Compared to the control group, the values of the mean thickness of alveolar septa increased only in the Hypo/Hyper group with values up to 9.92±5.10μm^-1^ without reaching significance (p<0.065) (Fig 3d), because of the high SD value in this group resulting from forming different subgroups. Thus, there were subgroups exhibiting some regions with no alterations in the septa, some with a moderate and some with a strong increase in septal thickness.

#### Morphology of Alveolar Epithelial cells type II (AEII)

**Fig 5:**
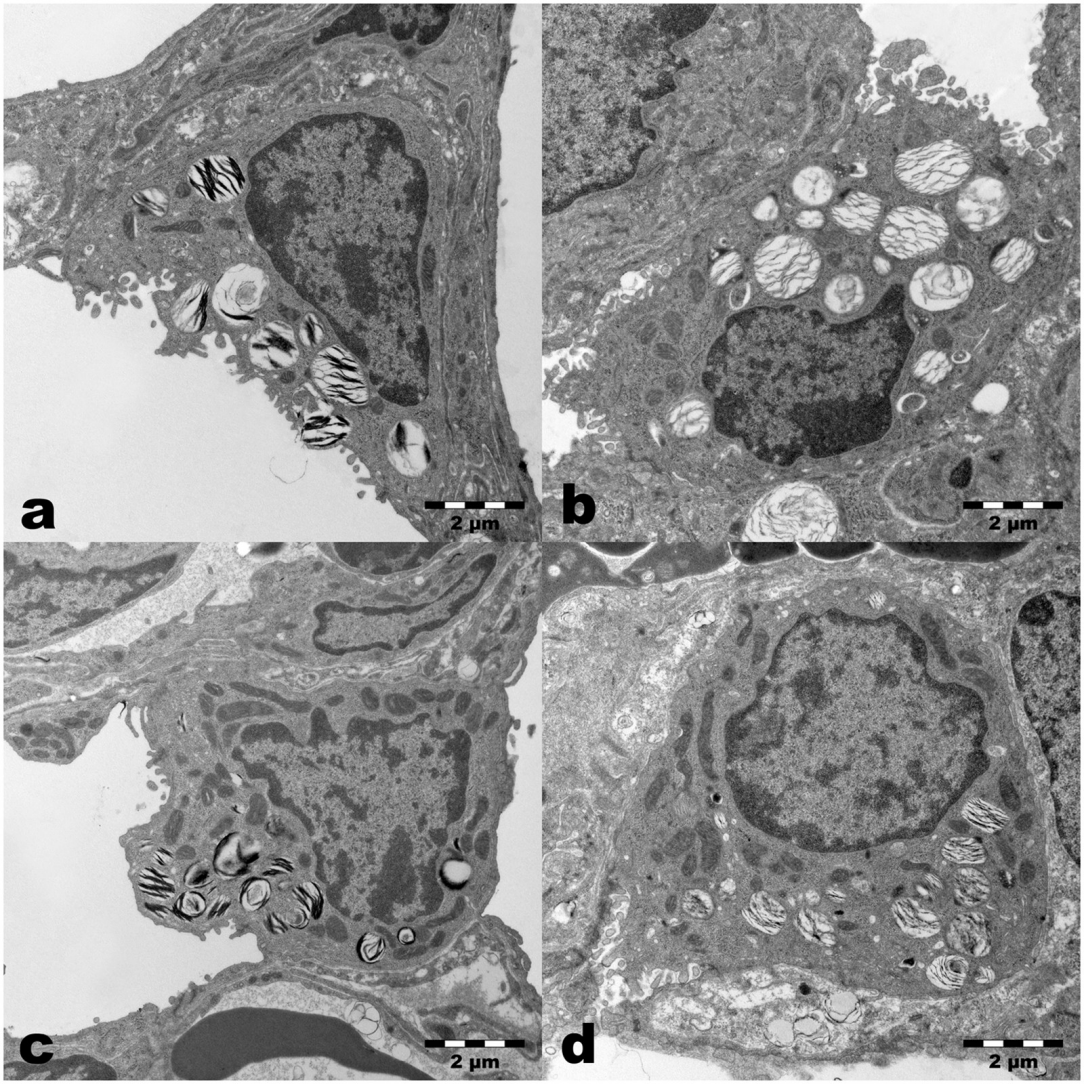
Ultrastructure of Alveolar Epithelial cells type II (AEII). a) Control lungs (No/No) showed AEII with numerous lamellar bodies of different size with more or less densely packed lipid lamellae and mitochondria with a dark matrix and densely packed cristae. b) After prenatal hypoxia (Hypo/No), in AEII normal distribution of subcellular components was seen. The partial loss of lipid lamellae in the Lb may be caused by longer fixation in aldehydes. c) After postnatal hyperoxia (No/Hyper) AEII exhibit numerous mitochondria and Lb without clearly visible differences compared to controls. d) After prenatal hypoxia and postnatal hyperoxia (Hypo/Hyper) AEII contain well preserved mitochondria and Lb with fixation-dependent partial loss of lamellae. Some smaller Lb sections are visible.

Looking at the electron microscopic level (Fig 5), structurally intact AEII locating predominantly in the niches of the alveolar septa were visible in all groups. The AEII contained numerous Lb of different size packed with lipid lamellae at different densities in controls. The number and size of Lb in the Hypo or Hyper groups looked similar. The Lb in the AEII of the Hypo/Hyper group seemed to be less numerous and notably smaller (Fig 5d). The partly lower degree of preservation of phospholipids in the Lb was found more or less frequently in all groups and is due to the duration of the fixation time in the aldehydes.

#### Volume densities of AEII were comparable, however, total volumes as well as mean volumes of AEII showed group specific-differences

**Fig 6:**
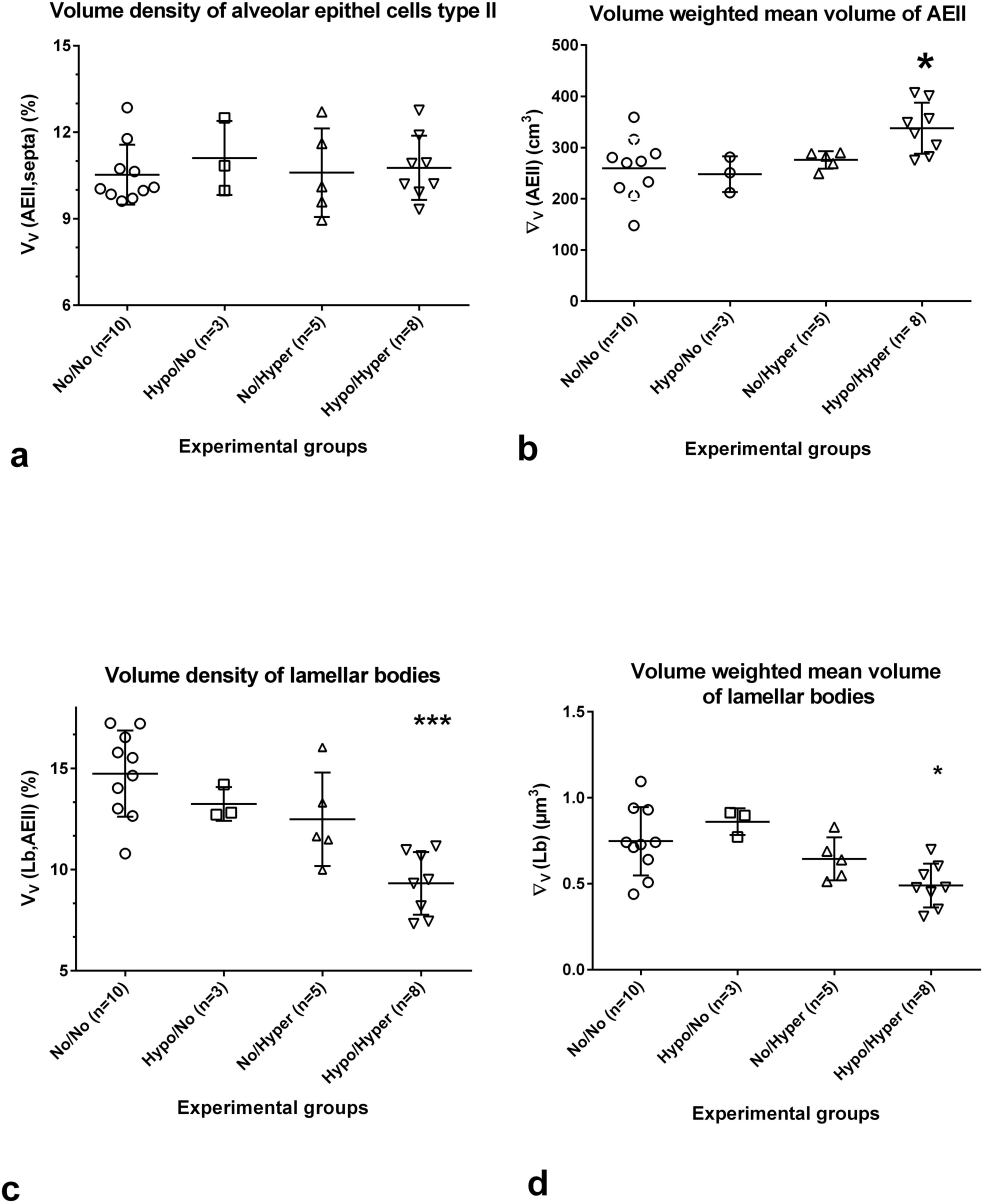
Stereological parameters characterizing Alveolar Epithelial cells type II (AEII) and lamellar bodies (Lb). Controls (normoxia group, No/No), after prenatal hypoxia and postnatal normoxia (Hypo/No group), after prenatal normoxia and postnatal hyperoxia ((No/Hyper group) and after prenatal hypoxia and postnatal hyperoxia (Hypo/Hyper group). Mean ± SD, *p<0.05, **p<0.01, ***p<0.001 compared to controls. a) The volume density of AEII showed a more or less pronounced variance in all groups. b) The volume weighted mean volume of AEII showed the significantly highest values compared to controls in the double hit model. c) Volume densities of Lb show comparable values in the one hit models, but significantly lower values in the Hypo/Hyper group. d) The volume weighted mean volume of Lb is comparable with control values in the Hypo/No and in the No/Hyper group. Significantly lower values were found in the double hit group.

The V_V_(AEII, septa) was 10.53±1.04% in the control group. Similar values were found in the experimental groups (Fig 6a). The total volume of AEII ((V(AEII, both lungs) was 24±2.5mm^3^ in control lungs (n = 8). No significant alterations were found in the experimental groups (Table 1). The volume weighted mean volume of AEII (∇_V_(AEII)) was 260±60cm^3^ (Fig 6b). While the ∇_V_(AEII) in lungs of the Hypo/No and No/Hyper was similar to controls, significantly higher values were found after prenatal hypoxia and postnatal hyperoxia (Fig 6b).

#### The mean volume of Lb was significantly reduced in the Hypo/Hyper group

**Fig 7:**
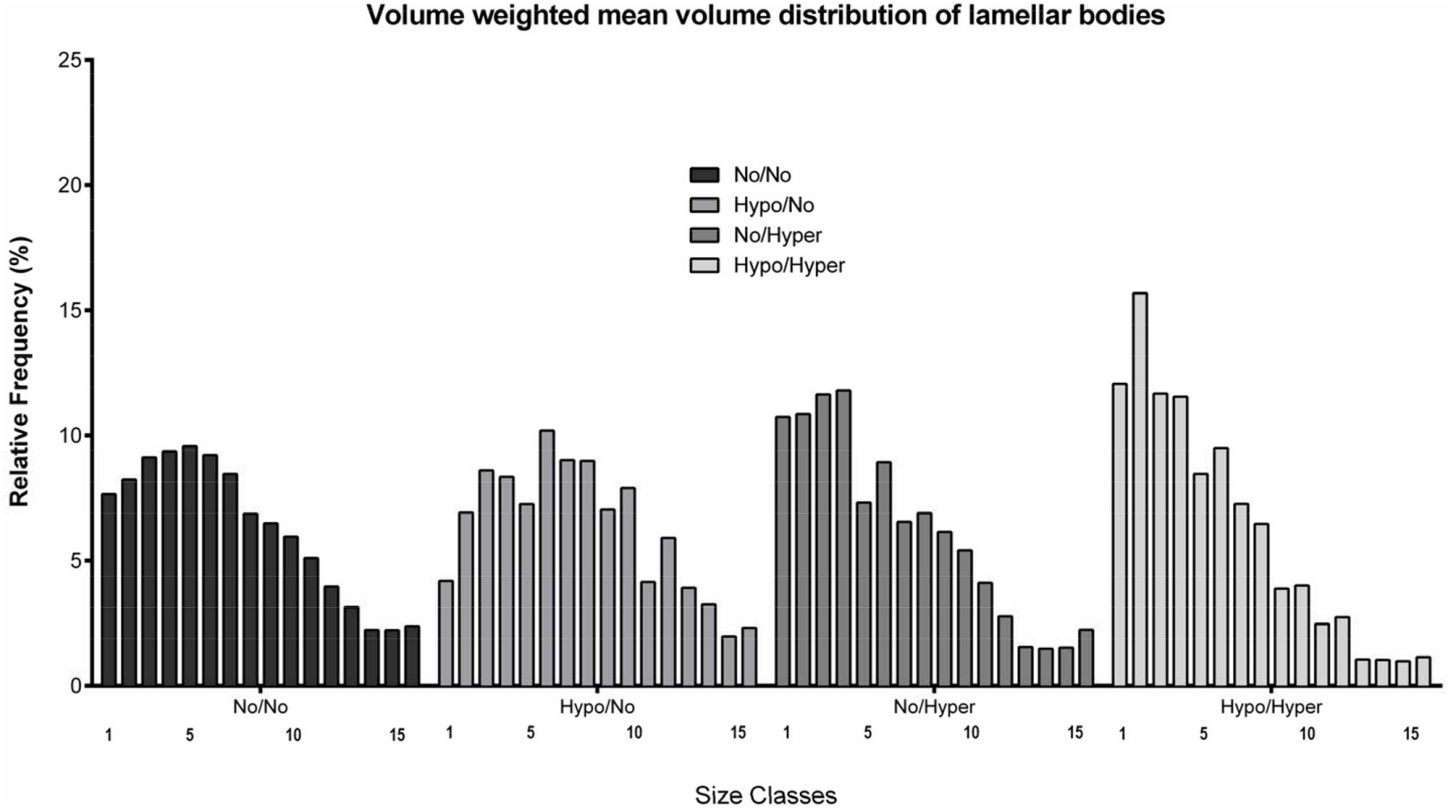
The relative frequencies of the distribution of Lb volume. The volume weighted mean volume was divided into 15 classes from the smallest to the largest volume. A pronounced shift to Lb with smaller volume was evaluated in the double hit group.

The intracellular volume density of Lb (V_V_(Lb, AEII)), the surfactant storage organelles, was 14.74±2.13% in controls (Fig 6c) leading to a total Lb volume per set of lungs (V(Lb, both lungs), n = 8) of 0.79±0.33mm^3^. The V_V_(Lb, AEII) exhibited group-specific differences (p<0.0013). Using multiple comparisons, V_V_(Lb, AEII) showed the significantly lowest values 9.32±1.6% in the Hypo/Hyper group (Fig 6c). The Lb volumes as percent of controls showed no significant alterations in the experimental groups (Table 1). The ∇_V_(Lb) was 0.75±0.20μm^3^ in controls (Fig 6d). The values differed significantly between the groups (p<0.0091). However, multiple comparisons between control values and values of the experimental groups led to significantly lower values only in the Hypo/Hyper group (0.49±0.13μm^3^, Fig 6d). The size distribution of Lb showed that in the Hypo/Hyper group significantly more Lb are located in the two smallest size classes compared to the other groups (Fig 7).

## Discussion

The aim of this study was to investigate the influence of different perinatal clinical risk factors on lung development in an established mouse model and to compare them with respect to their suitability for studying BPD.

### Prenatal hypoxia and postnatal normoxia

The model of pure prenatal hypoxia used is representative of the stage of lung development in very preterm infants. As clearly shown, 14 day old mice suffering from prenatal hypoxia at an inspired oxygen fraction of 0.1 exhibited no retardation of morphological lung development. However, a significantly lower lung volume compared to controls was determined. Using the same mouse model, Gortner et al. likewise found no morphological retardation of lung development immediately at the end of hypoxia on gestational day (gd) 18.0, but a significantly lower body weight [35], which still remained on postnatal day 1 [25]. Thus, our Hypo/No model indicates significant growth restriction without alterations in lung architecture. Further authors described differential influence of prenatal hypoxia using a rat model. A significant reduction in fetal growth and lung weight was found within the canalicular phase after intermittent exposure to 10% oxygen for 2h between gd 15 and 19 [49]. Also significantly lower lung volumes and body weights on gd 21 were evaluated in rat fetuses after intrauterine hypoxia with 10% oxygen supply between gestation day 14 and 21 [50]. On the other hand rat pups of mothers kept in a hypoxic atmosphere of 13% O_2_ 3 weeks before, throughout and after the whole pregnancy exhibited an attenuation of septation combined with a reduction of alveolar number on postnatal day 14 at the end of alveolarization [51]. Furthermore, after a short prenatal hypoxia (10% O_2_) between gd 16.5 and 17.5 the fetuses remained in the canalicular stage after one day of normoxia, while normoxic controls reached the saccular phase [52]. With regard to the surfactant system, Gortner et al described a down-regulation of gene expression of the surfactant proteins (SP) SP-A, B, and C immediately after the end of prenatal hypoxia of 10% on gd 17.5. They suggest a potentially hypoxia-induced delay in lung maturation concerning the surfactant system [35]. However, our results showed that at the end of the postnatal alveolarization phase the surfactant system had recovered from prenatal hypoxia. Volume, size and density of AEII as well as of the Lb did not differ compared to controls. Therefore, we suggest a normal expression of SP, predominantly, the hydrophobic SP-B, which is necessary for the formation of Lb [53,54]. Summarizing all data, the influence of pure hypoxia is less successful to investigate retardation of lung development [23], because normal lung development occurs in relative hypoxic conditions in utero, which are beneficial for development [55]. Hypoxia greatly stimulates the expression of vascular endothelial growth factor (VEGF), a powerful inducer of vasculogenesis [56]. VEGF genes as well as other hypoxia-dependent genes are probably activated by hypoxia-induced factors HIF-1/2α [57]. Therefore, the hypoxia-induced intrauterine growth retardation [35,49,58] is not necessarily associated with retardation in lung maturation. Thus, the influence of prenatal hypoxia on lung maturation depends on the prenatal application time, the duration of exposure, the oxygen tension as well as the time period of normoxia following hypoxia.

### Prenatal normoxia and postnatal hyperoxia

Prenatal normoxia and postnatal hyperoxia with 75% oxygen exposure from postnatal day 1 to 14 led to signs of morphological lung immaturity as seen in a significantly reduced total septal surface, which is an indirect measure for septation and also for alveolarization. Significantly reduced total parenchymal airspace volume may be a result of a significantly decreased lung volume. However, unlike other authors we found no significant increase in the septal thickness, and size of parenchymal airspaces using the point sampled intercept method. A significant increase of alveolar size on postnatal day 14 after short term hyperoxia (postnatal day 1 to 4) with 90% O_2_ was determined in mice by mean alveolar size and mean linear intercept [59]. Also, postnatal exposure of newborn rats to 95% for 14 days or to 65% oxygen for 7 days led to a significant increase of mean linear intercept and mean alveolar volume as an indication of an arrest in alveolarization [60,61]. A significant increase of airspace measured by mean linear intercepts or radial alveolar counts was also achieved after a postnatal exposure of neonatal mice to 65% or 60% oxygen for seven days [62,63]. Chronical hyperoxia models with 80 or 85% oxygen exposure for 28 days also resulted in enlarged alveoli, partly with increased septal wall thickness [22,64,65]. Thus, the degree of BPD-like delay in lung development depends on the oxygen dose, the administration period and the timing as well as on the morphometric parameters. We used the point-sampled intercept method to determine the ‘‘volume-weighted” mean parenchymal airspace volume as a measure for the size of airspaces and size heterogeneity [46]. This technique gives information about the mean volume and the variability of size of the specified parenchymal spaces which were defined as single particles [46]. Because it is not possible to distinguish without 3-D between saccules and alveoli, and it is quite difficult to differentiate between ductus and enlarged sacculi, we decided to determine the alveoli, sacculi and ductus as parenchymal airspaces. Although we determined a significant decrease in the septal surface as one hint for disturbances in alveolarization the used parameter characterizing alveolar enlargement did not significantly increase (p<0.13). On the one hand possibly be that this parameter is less sensitive than other parameters such as mean linear intercept or radial alveolar counts used for determining alveolar size after hyperoxia, on the other hand the interindividual variation could prevent significance. So one of the five pups used in this group showed no increase in airspace volume (Fig 4). Not least, our stereological methods taken into account the whole lung reveal that the portion of enlarged airspaces was not sufficient for significant changes.

Looking at the surfactant system the total volumes of AEII and Lb were not significantly affected by hyperoxia. Furthermore, no structural differences of AEII were found. Studies on adult rats have shown that after 48h of hyperoxia and after 60h of hyperoxia Lb were significantly smaller [32,66]. A lower sensitivity of pups may not be ruled out.

### Prenatal hypoxia and postnatal hyperoxia and other double hit models

Using higher animal numbers and further stereological parameters, we could confirm the already published results [25,36]. Summing up our results, the alterations are comparable to the morphological signs of BPD. As described before, this model also leads to significant growth restriction (decreased total body length, decreased lung and brain weight) by postnatal day 14 [23,25,36]. The prenatal hypoxia mimics intrauterine growth restriction, which is seen in many preterm infants [67]. Large observational clinical trials in different ethnic populations constantly show an association of IUGR-SGA (intrauterine growth retardation-small for gestation age) status and an increased risk for BPD [67–70]. IUGR resulting from placental pathology causes intrauterine hypoxemia, which is a risk factor for BPD [35]. SGA-fetus does pass from intrauterine hypoxemia to a relative hyperoxia reinforced by postnatal ventilation with elevated O_2_ concentrations [23,71] as imitated in our model. The amplification effect of developmental retardation is obvious and furthermore this model is closer to the clinical events [23] corresponding to the feature of the so called “new” BPD [19,25]. The established Hypo/Hyper model, not used by other authors until now, is one of numerous different so called double hit models to take the multifactorial etiology of BPD into account and to trigger a severe BPD-like lung disease in newborn mice [72–75]. Instead of perinatal hypoxia another possibility is to induce perinatal inflammation as a trigger. Perinatal LPS at various times, doses and duration and postnatal hyperoxia with various FiO_2_ values resulted in a differently pronounced arrest in alveolar development, depending on duration of exposure and O_2_ concentration [73–76]. LPS exposure as well as hypoxia alone may not be sufficient to disturb lung development [75,76]. In our model we did not find any signs of inflammation in histology indicating no or only minor inflammation. These results are in accordance with earlier investigations using the same model finding no significant elevation of inflammatory cytokines in mice [36]. Our results underline out the fact that the so called new type of BPD, is supposed to imitate generally less pronounced inflammation [18,77].

Looking at the surfactant system, we determined a significant increase in the mean volume of the AEII, probably by swelling or immaturity. However, the values of V_V_(AEII, septa) as well as of the total volume of AEII did not differ. The significant increase of the ∇_V_(AEII) is combined with a significant decrease in the V_V_(Lb, AEII), suggesting the phenomenon of a reference trap [40], but due to the significant decrease of ∇_V_(Lb) the occurrence of smaller Lb may be responsible for the decreased V_V_(Lb, AEII) values. These results show that the surfactant system is also affected, which confirms our earlier results [25,36]. AEII are the primary targets of the hyperoxia-induced lung injury [78]. In adults a 48h or 60h exposure to 98% oxygen led to a decrease in Lb size and number [32,66]. We could show here the perinatal influence of oxygen concentration on the size distribution of Lb as a sign of immaturity [39]. We showed further that the downregulation of SP-C correlates with a decrease in the V_V_Lb [25,36].

Thus, exposure to postnatal hyperoxia (75% oxygen) with or without a prenatal hit does influence morphological lung development [71]. However, the relative gene expression of elastin, one of the numerous factors, which are jointly responsible for normal alveolarization [64,79] did not change significantly in our double hit model [25]. Relative hyperoxia following premature birth may lead to a reduction of the HIF-α, and so to decreased levels of VEGF, resulting in an interruption of vascularization, but also of alveolar formation [80]. Vascularization may drive alveolar development [71,81] and may be a key mechanism for the decreased alveolarization in premature infants resulting in BPD [71,82]. VEGF gene and protein expression in the Hypo/Hyper group was comparable with that of controls as shown earlier [25]. Other signaling systems involved in vascularization and alveolarization such as the LPR5/Tie2/Ang signaling system (Lipoprotein receptor-related protein 5, angiopoietin and its receptor Tie2) may also be influenced by hyperoxia [83]. However, the main focus of this investigation was to determine the alveolar formation and the intracellular surfactant system using stereological methods. Characterizing the capillary network with accuracy, bias and precision without systematic error using stereological methods requires that the profiles of capillaries are clearly recognizable. Therefore, perfusion fixation would be the method of choice [84,85]. Regrettably all lungs in the present study were fixed by instillation. A new experimental approach using perfusion fixation of lungs from all groups will be necessary to establish suitable stereological methods to determine differences in vascularization in the different oxygen supply models.
